# Small + Safe + Well: lessons learned from a Total Worker Health® randomized intervention to promote organizational change in small business

**DOI:** 10.1186/s12889-022-13435-y

**Published:** 2022-05-24

**Authors:** Natalie V. Schwatka, Miranda Dally, Erin Shore, Liliana Tenney, Carol E. Brown, Joshua G. Scott, Lynn Dexter, Lee S. Newman

**Affiliations:** 1 Center for Health, Work & Environment, Colorado School of Public Health, Anschutz Medical Campus, University of Colorado, 13001 E. 17th Pl., 3rd Floor, Mail Stop B119 HSC, Aurora, CO 80045 USA; 2 Department of Environmental and Occupational Health, Colorado School of Public Health, Anschutz Medical Campus, University of Colorado, 13001 E. 17th Pl., 3rd Floor, Mail Stop B119 HSC, Aurora, CO 80045 USA; 3 Present Address: University of North Carolina at Chapel Hill, Chapel Hill, NC USA; 4 Present Address: 2U, Inc., Lanham, MD USA; 5 Department of Epidemiology, Colorado School of Public Health, and Department of Medicine, School of Medicine, Anschutz Medical Campus, University of Colorado, 13001 E. 17th Pl., 3rd Floor, Mail Stop B119 HSC, Aurora, CO 80045 USA

**Keywords:** Occupational health and safety, Training, Implementation science, Employer health promotion, Organizational leadership, Total worker health, Organizational behavior, Workplace health

## Abstract

**Background:**

Leadership commitment to worker safety and health is one of the most important factors when organizations develop and implement a *Total Worker Health*® approach. We aimed to assess the effectiveness of a *Total Worker Health* (“TWH”) leadership development program that targeted owners and other senior-level leadership positions on changing organizational and worker outcomes from baseline to one-year later.

**Methods:**

The Small + Safe + Well study included small businesses from a variety of industries in the state of Colorado, USA that were participating in Health Links™. We designed a randomized waitlisted control comparison design (RCT) to evaluate the added benefit of a TWH leadership development program. An employer assessment tool was used to assess TWH policies and programs, and an employee health and safety survey was used to assess safety leadership and health leadership practices, safety climate and health climate, safety behaviors and health behaviors, and well-being. We used a linear mixed model framework with random effects for business and employee to assess the impact of intervention on the outcomes of interest.

**Results:**

Thirty-six businesses (37% retention) and 250 employees (9% retention) met the RCT study inclusion criteria and were included in the analysis. Businesses improved their TWH policies and programs score from baseline to one-year later, regardless of leadership intervention group assignment. Neither intervention group demonstrated improvements in employee-reported outcomes.

**Conclusions:**

This study sought to address a gap in the literature regarding small business senior leadership development for TWH. Our study demonstrates many of the challenges of conducting studies focused on organizational change in workplaces, specifically in small businesses. When designing TWH intervention studies, researchers should consider how to best engage small business leaders in interventions and implementations early on, as well as methods that are well matched to measuring primary and secondary outcomes longitudinally. Future research is needed to test the feasibility and sustainability of TWH interventions in small business.

**Trial registration:**

The trial was retrospectively registered with ClinicalTrials.gov (ID U19OH011227).

**Supplementary Information:**

The online version contains supplementary material available at 10.1186/s12889-022-13435-y.

## Background

Leadership commitment to worker safety and health is one of the most important factors when organizations develop and implement a *Total Worker Health*® approach as conceptualized by the United States’ National Institute for Occupational Safety and Health (NIOSH) [1–4]. *Total Worker Health* (“TWH”) is defined as ‘‘policies, programs, and practices that integrate protection from work-related safety and health hazards with promotion of injury and illness prevention efforts to advance worker well-being’’ [5]. The approach emphasizes that work is a social determinant of health and therefore focuses on changing working conditions to protect and promote workforce health. Leaders play a major role in helping organizations change the way they address workforce safety and health. They can drive the development and implementation of TWH in practice. Furthermore, the use of leadership skills by those in formal leadership roles are directly related to positive workforce health, safety, and well-being outcomes [6]. In previous publications [4, 7] we describe our theoretical model for how leadership impacts workforce health. We discuss this briefly below while considering current TWH leadership research. Ultimately, in this paper we describe the evaluation of a TWH leadership intervention for small business leaders.

We propose that workers health, safety, and well-being are ultimately the product of their working environment, suggesting that TWH interventions need to target working conditions. Like other TWH researchers [8], we draw upon Burke and Signal’s multi-level model of safety [9] and the socio-ecological model to guide the development of our TWH intervention. Each organization has its own espoused values, leadership strategies, and policies and programs that set the stage for how healthy and safe workers can be on the job. Sorensen et al. aptly noted that the “workplace acts as both an accelerator and preventor” of injury and illness and that “workers may perceive changes in their individual health behaviors to be futile in the face of” working conditions that make it challenging to protect and promote their health [10]. These organizational level factors influence worker outcomes related to motivation, knowledge, behavior, and injury and illnesses. Leadership practices of those in a formal leadership role represent a key target for interventions that address working conditions.

Second, we propose that for interventions to result in improved working conditions, they must address both transactional and transformational organizational changes [11]. Transactional changes reflect changes in organizational structures and systems (policies and practices) that support implementation of the TWH approach. Transformational changes reflect changes to leadership support, business mission and strategy, a supportive organizational culture and employee level empowerment. Previous TWH interventions commonly address transformational change components, such as employee participatory methods to identify and implement a TWH approach [12, 13]. Also, while some TWH interventions focus on transformational intervention factors around management participation, few seek to develop leadership capacity for TWH by educating and guiding leaders in implementing a TWH approach with their business, and none focus on doing so amongst small business owners or other senior-level decision-makers [4, 7, 8, 14]. In this study, we posit that small businesses must undergo organizational changes to effect change in workforce health, safety, and well-being outcomes.

Recently, a few industry specific TWH interventions that include a supervisory training or consultation component have been tested and have yielded mixed results. Researchers have used a variety of means to engage supervisors, including online training and behavior tracking [15–17], in-person intervention awareness training [18, 19], and inclusion in a TWH worker-management design team [12, 13]. These interventions included transformational change components, such as methods to support employees’ work/life balance [17]. However, the applicability of these interventions to small businesses remains unknown. Small businesses typically have less of a systematic approach to workplace health and safety, common barriers may include lower levels of management commitment and employee engagement as well as a lack of resources for program implementation [20–22]. Given that about half of Americans are employed in small businesses, defined as fewer than 500 employees, there is a need to design and test TWH interventions that reach senior-level decision-makers in these settings.

In the small business world, senior-level decision-makers are especially important in driving the organizational culture. Because the field of TWH embraces systems-focused methods and approaches [23–26], a better understanding of how to impact business system decision-makers will be essential. Our research, and that of others, suggests that small business owners and other senior leaders are a key target audience for interventions as their actions are significantly related to business and employee TWH practices [27–30]. While TWH interventions with leadership components hold promise as methods to change organizational behavior with ultimate effects on workforce outcomes [16, 18], the interventions have been traditionally focused on supervisor level leadership behaviors. Supervisors are key to the implementation of the TWH approach on a day-to-day basis. It is important to also focus on the TWH leadership of owners and other senior leaders as they play a critical role in including TWH in the business mission and vision, allocating resources for TWH, and are key role models for TWH practices.

The Small + Safe + Well (SSWell) study (2015–2020) conducted by researchers at the Center for Health, Work, & Environment, a NIOSH TWH Center of Excellence, recruited 132 small businesses in the state of Colorado to assess the effectiveness of a TWH leadership development program, described below, that targeted owners and other senior-level leadership positions. Below we examine our hypotheses that small businesses that participate in a TWH leadership development intervention for owners and other senior-level leadership positions will demonstrate more positive change in their (H1) business TWH policies and programs and (H2) employee reported safety leadership and health leadership practices, and that their workers will report better (H3) safety climate and health climate perceptions, (H4) safety and health behaviors and (H5) self-reported well-being from baseline to one-year later, compared to businesses whose leaders did not participate in a TWH leadership development program. We conclude with a brief discussion on the challenges of recruiting and retaining many small businesses and their senior leaders for a large-scale randomized trial.

## Methods

All study methods were approved by the Colorado Multiple Institutional Review Board. The trial was retrospectively registered with ClinicalTrials.gov on 16/07/2021 (ID NCT04965415). It was conducted in accordance with CONSORT guidelines. Details on the study’s theoretical background, study sample, and cross-sectional relationships have been published [4, 22, 27, 28, 31, 32].

### Control – usual practice

Businesses in the control group participated in Health Links® [33, 34]. The purpose of the Health Links program is to help the business develop TWH policies and programs (transactional activities) that are relevant to their business and workforce. On a yearly basis, businesses complete an online Healthy Workplace Assessment and receive a score card that benchmarks their TWH policies and programs. Businesses that complete the Healthy Workplace Assessment are awarded recognition at one of three levels based in the results and are offered consultation sessions (up to two) with a Health Links advisor to interpret their scores and set goals for the coming year. Typically, one person per business or a health and safety team completes these activities on behalf of the business.

### Intervention

The businesses in the intervention group participated in Health Links as well as a TWH leadership development program. The TWH leadership development program included in-person and virtual components based on validated leadership theories and best practices (transformational activities). Full details on the TWH leadership development program have been published previously [22]. In brief, the program focused on helping the small business leader make transformational and transactional changes to their organization’s TWH practices. The program facilitated transactional change by educating leaders about their businesses current TWH business policies and programs. For example, leaders assessed their current safety policies and programs. Additionally, the program helped the leader understand their business’s current culture as it pertained to TWH and how they could leverage leadership practices (e.g., role modeling) to enhance their culture to facilitate transformational changes. For example, after reviewing their current TWH policies and programs, the leaders reviewed the results of their organization’s employee health and safety survey considering their current TWH policies and programs. Oftentimes leaders observed a disconnect between their businesses TWH strategy and what their employees thought about the health and safety conditions of their organization.

The program was offered to a senior leader as well as one additional member from the organization, usually a safety manager or human resource manager. Participants first completed a pre-training survey that assessed their current TWH leadership practices and personal health, and were asked to reflect on their businesses current approach to TWH. During the six-hour in-person training, leaders participated in a training focused on helping them create three goals to work on over the next three months: 1) a transactional goal around their business’s TWH strategy to facilitate changes to their business TWH policies and programs, 2) a transformational goal around their employees’ perceptions of a healthy and safe workplace to facilitate changes to their culture of health and safety, and 3) a goal for their personal health to facilitate leaders’ ability to role model and maintain their health while facilitating organizational changes. During this training, leaders’ reviewed data on their businesses current approach to TWH, including their Healthy Workplace Assessment™, employee health and safety survey, and the leader’s own responses to their pre-training survey. In the three-months after this training, participants engaged in two training transfer activities to help them meet their goals: 1) up to three 30-min one-on-one coaching sessions and 2) an online, social goal tracking platform. We customized the www.stickk.com goal setting platform to allow leaders to create goals, select accountability methods, and track goal progress. Leaders could choose to add a disincentive for not meeting their goal, to invite others to support them, and to receive support from trainers.

### Participants

The SSWell study was designed to be a randomized TWH intervention study of small businesses (< 500 employees) that participated in Health Links [33, 34]. All businesses were located in the state of Colorado in the United States. Businesses were recruited at the organizational level. Once enrolled, all employees at each organization were invited to participate in the assessments used to evaluate the intervention. Enrollment was open from April 2017 to August 2019.

**Fig. 1:**
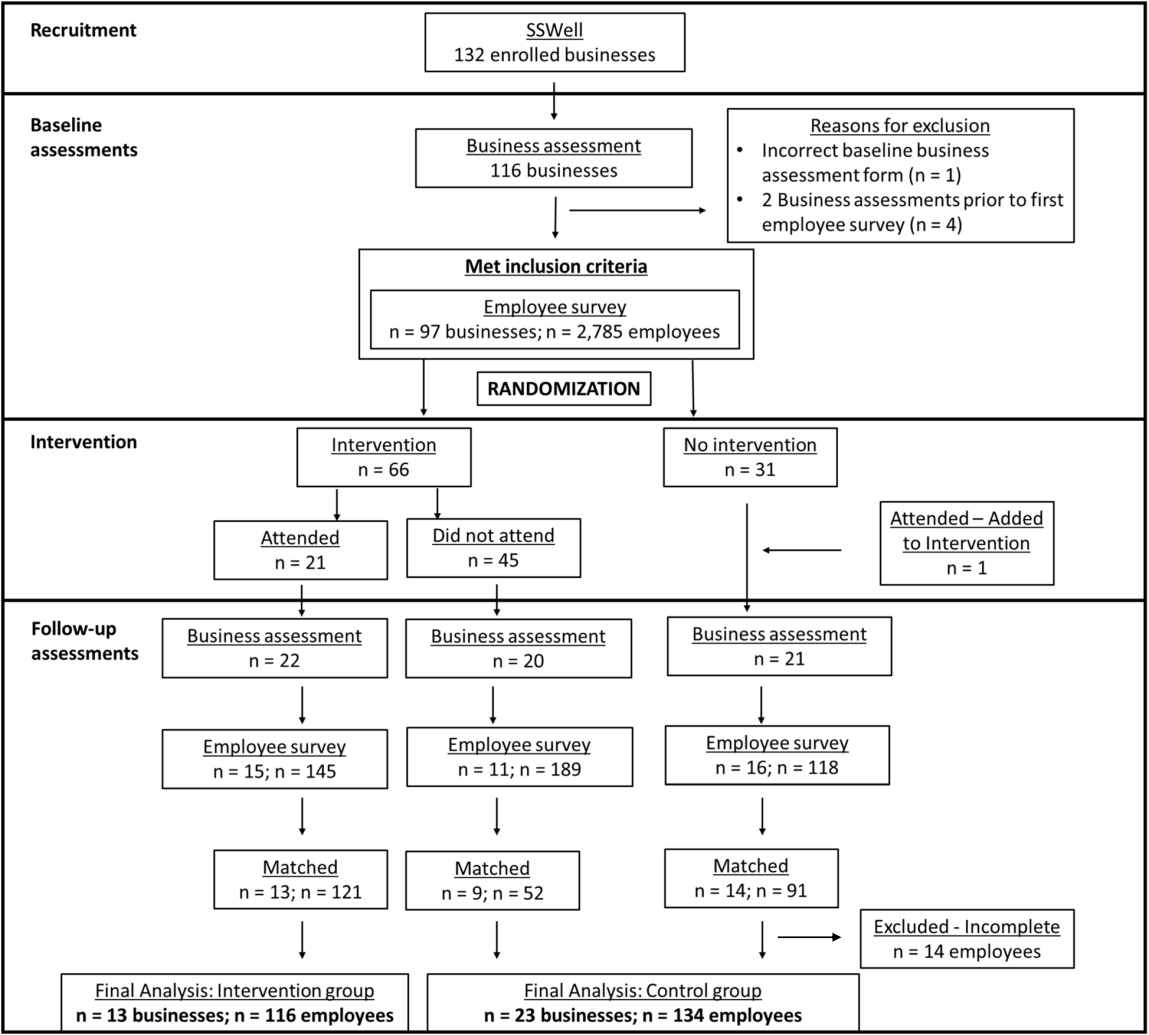
Study design for the SSWell waitlisted control comparison design study representing the 36 unique organizations and 250 of their employees participating as treated

We designed the study with the intention of conducting a randomized waitlisted control comparison. Businesses were assigned to either the early intervention arm occurring during the first year of their study participation or the lagged intervention arm occurring during the second year of their study participation. Thus, all businesses had the opportunity to participate in the TWH leadership development program intervention. We randomly assigned businesses into either the early or lagged intervention arm using a 2:1 randomization schema. The sequence of randomization was generated in 42 blocks of 12 prior to the start of the study. The randomization list was maintained by study personnel not involved in the recruitment or intervention implementation. As illustrated in Fig. 1, some businesses that were enrolled in the early arm did not participate in the intervention until their second year due to scheduling constraints. Similarly, some businesses that were offered the intervention chose not to participate in the intervention. We performed the analysis on the businesses as treated.

An overview of the study design and number of participating businesses and employees are summarized in Fig. 1. To be included in the present analysis, businesses must have completed both first- and second-year assessments. Additionally, employees must have completed both the first- and second-year surveys while employed at the same business. This was confirmed by matching individual responses within businesses based on the unique identifier. All individuals who matched were included in all individual analyses. All businesses that had at least one employee participating at both time points were included in business level analyses.

Ninety-seven businesses enrolled and met inclusion criteria. During follow-up, 63 businesses completed the business assessment (57%) with 42 of these businesses having had at least 1 employee complete the follow-up survey (38%). The final business cohort consisted of 36 businesses that had at least one matched employee complete both the initial and follow-up survey, which represents 37% of the baseline business sample. There were 2,785 baseline employee surveys completed and 452 employees completed follow-up surveys. However, only 264 employees completed both the initial survey and the follow-up survey. Of these, 14 surveys were missing data on our outcomes of interest and were excluded from the final cohort. The final employee cohort consisted of 250 employees who completed both the initial and the follow-up surveys, which represents 9% of the baseline employee sample.

### Data collection and measures

Measurements were collected at two time points approximately one year apart via an online business assessment and online employee survey. Upon enrollment, businesses completed the online Healthy Workplace Assessment. The study coordinator then worked with each organization to distribute an employee health and safety survey to all employees through REDCap (Research Electronic Data Capture) [35]. The employee health and safety survey was made available in both English and Spanish. No individual identifying information was collected. Participants were asked to fill in a unique identifier comprised of the second letter of the first name, the first letter of the city of birth, and the last two digits of the social security number. Individuals who filled out the employee health and safety survey were asked if they wanted to be entered into a drawing to receive a $100 gift card. Those who did were taken to a separate survey where they entered a contact email address. Participating businesses completed the Healthy Workplace Assessment and their employees completed the employee health and safety survey on an annual basis until completion of the SSWell study in July 2020. For this study, we utilized data from baseline and year one assessments.

### Outcomes

We evaluated three primary outcomes: 1) business-reported TWH policies and programs; 2) employee-reported safety leadership practices; and 3) employee-reported health leadership practices. The Healthy Workplace Assessment measured transactional activities reflecting organizational level TWH policies and programs through six benchmarks: 1) organizational supports, 2) workplace assessments, 3) health policies and programs, 4) safety policies and programs, 5) engagement, and 6) evaluation. The TWH policies and programs score is the sum of these six benchmarks and has been validated through formative research, including focus groups, and using a verification checklist at the time of advising that helps assess the accuracy of answers and confirm specific organizational behaviors [34]. The total possible score is 97 with 0 reflecting no TWH policies and programs and 97 representing significant levels of TWH policies and programs. More detail on the assessment can be found in Tenney et al. [33]. We developed the safety leadership (5 items, α = 0.89) and health leadership (5 items, α = 0.92) employee survey questions based on the leadership questions that were asked in the Healthy Workplace Assessment, including leaders’ communication, role modeling, employee recognition, resource allocation, and accountability. One example item is ‘‘Leaders are role models for prioritizing safety.’’ Thus, these leadership constructs reflect the practices that small business leaders engage in to actively role model and engage their workforce in health and safety efforts. Its reliability and validity have been demonstrated [27]. Both variables were measured on a 1–5 Likert scale from strongly disagree to strongly agree.

We also measured secondary outcomes using the employee health and safety survey. These items were measured on a 1–5 Likert scale from strongly disagree to strongly agree. We used Lee et al.’s [36] 6-item organizational commitment to safety scale to assess safety climate (α = 0.90) and an abbreviated version of Zweber et al.’s [37] health climate scale. We used their 4-item organizational commitment to health and well-being factor (α = 0.81). An example item is, ‘‘When management learns that something about our work or the workplace is having a bad effect on employee health or well-being, then something is done about it.’’ Thus, health and safety climates reflect employees’ perceptions of their organization’s commitment to workplace health and safety programs and their beliefs about how much their organization values a healthy and safe workplace. Health behaviors (α = 0.93) and safety behaviors (α = 0.87) were measured using items from Griffin & Neal and reflect proactive participation in health and safety programs, respectively [38]. An example question is, ‘‘I promote the safety program within the organization.’’ Thus, behaviors reflect behaviors that help the organization develop working conditions that support health and safety. Well-being was measured using Staehr et al.’s [39] 5-item scale (α = 0.84). All these measures have been found to be reliable and valid [27, 28, 40]. All employee survey constructs represent the average response to each of the questions used to measure the construct.

### Primary predictor

Our primary predictor was participation in the TWH leadership development program. We created intervention groups based on the observed timing of participation in the TWH leadership development program. The businesses in the intervention group participated in TWH leadership development program between their baseline- and first-year participation in the SSWell study. The control group consisted of businesses that either did not participate in the TWH leadership program or that participated after the study period of interest.

### Statistical analysis

We used ANOVA, Chi-squared, or Fisher’s Exact tests to test for baseline differences between intervention groups, as well as baseline differences between those who participated in the study and those who were lost to follow-up, as appropriate. The normality of all outcome variables was visually assessed.

We used a linear mixed model framework with random effects for business and employee to assess the impact of intervention on the outcomes of interest. Random effects for employee were excluded from the TWH policies and programs score model. To test whether the intervention effect differed between businesses that received the TWH leadership development program and those that did not we included an intervention group by time effect. Least square means were used to determine the point estimate and 95% confidence interval (95%CI) for time specific intervention group estimates while contrasts were used for between intervention group comparisons.

In all models, we adjusted for potential confounding effects of timing of study events. Covariates were added for time between training and follow-up Healthy Workplace Assessment and employee health and safety survey as well as time between baseline and follow-up Healthy Workplace Assessment and employee health and safety survey. Alpha levels were set at 0.01 for our hypothesis tests to account for multiple comparisons. All analyses were conducted using SAS version 9.4 (Cary, NC).

## Results

Figure 1 provides retention rates for each level of the study. Of the 97 businesses that enrolled and met the inclusion criteria, 36 businesses (37%) were included in the analysis. Similarly, of the 2,785 employees who filled out an initial employee health and safety survey, 250 employees (9%) were included in the analysis. The 36 participating businesses significantly differed from the 61 excluded businesses based on business size and baseline TWH policies and programs score. On average, businesses that were included had an average of 110 employees (SD: 113) compared to 61 employees (SD: 75) at businesses that were excluded (*p*-value: 0.014). Businesses that were included had an average baseline TWH policies and programs score of 46 (SD: 18) compared to 37 (SD: 14) for those that were excluded (p-value: 0.005). All baseline comparisons between included and excluded businesses can be found in Additional file 1.

The 36 participating businesses represented a variety of industries with 28% from the health care and social assistance sector (*n* = 10). Most of the businesses had between 11 to 200 employees, (mean 110 [SD: 113; Range: 4 – 430]). Over 20% (*n* = 8) of the participating businesses operated in rural areas. There were no statistically significant differences based on industry, size, location, or baseline TWH policies and programs score between businesses participating in intervention and those that did not (Tables 1 and 2).

**Table 1:** Baseline business characteristics and employee demographics overall and by intervention group

**Business characteristics**	**Overall (*n* = 36)**	**Intervention (*n* = 13)**	**Control (*n* = 23)**	***p*-value**
*Industry*				0.527
Construction	3 (8%)	0 (0%)	3 (13%)	
Education	3 (8%)	2 (15%)	1 (4%)	
Health Care/Social Assistance	10 (28%)	4 (31%)	6 (26%)	
Public Administration	2 (6%)	0 (0%)	2 (9%)	
Other	18 (50%)	7 (54%)	11 (48%)	
*Size*				0.081
Micro (2–10)	3 (8%)	0 (0%)	3 (13%)	
Small (11–50)	13 (36%)	6 (46%)	7 (30%)	
Medium (51–200)	14 (39%)	7 (54%)	7 (30%)	
Large (201–500)	6 (17%)	0 (0%)	6 (26%)	
Number of employees		78 (54)	127 (133)	0.209
Urban	28 (78%)	10 (77%)	18 (78%)	0.999
**Employee Demographics**	**Overall (*n* = 250)**	**Intervention (*n* = 116)**	**Control (*n* = 134)**	***p*-value**
Age	41.7 (11.9)	40.8 (11.7)	42.4 (12.2)	0.329
Gender				0.999
Male	63 (25%)	29 (25%)	34 (25%)	
Female	186 (74%)	87 (75%)	99 (74%)	
Race/ethnicity				0.362
White, non-Hispanic	217 (87%)	99 (85%)	118 (88%)	
Hispanic/Latino/Spanish Origin	24 (10%)	11 (9%)	13 (10%)	
Other or did not provide^a^	9 (5%)	6 (6%)	3 (2%)	
Job level				0.642
Manager	92 (37%)	41 (35%)	51 (38%)	
Non-manager	157 (63%)	74 (64%)	83 (62%)	
Did not provide	1 (1%)	1 (1%)	0 (0%)	
Tenure, years	5.4 (5.9)	4.9 (4.9)	5.8 (6.6)	0.215
Education				0.062
Did not complete high school	0 (0%)	0 (0%)	0 (0%)	
High school/GED	20 (8%)	5 (4%)	15 (11%)	
Some college/2-year degree	64 (26%)	28 (24%)	36 (27%)	
4-year college degree	107 (43%)	48 (41%)	59 (44%)	
Graduate/professional degree	54 (22%)	33 (29%)	21 (16%)	
Did not provide	5 (2%)	2 (2%)	3 (2%)	

^a^ Detailed numbers omitted from table in the interest of maintaining participant anonymity

**Table 2:** Baseline scores on all study outcomes overall and by intervention group

**Business**	**Overall (*n* = 36)**	**Intervention (*n* = 13)**	**Control (*n* = 23)**	***p*-value**
TWH policies and programs score	46.3 (18.1)	45.1 (19.2)	47.0 (17.9)	0.77
**Employee**	**Overall (*n* = 250)**^**a**^	**Intervention (*n* = 116)**	**Control (*n* = 134)**	***p*-value**
Safety Leadership	3.68 (0.76)	3.76 (0.76)	3.61 (0.76)	0.103
Health Leadership	3.52 (0.86)	3.45 (0.89)	3.57 (0.84)	0.281
Safety climate	3.83 (0.76)	3.88 (0.70)	3.78 (0.80)	0.293
Health climate	3.91 (0.70)	3.87 (0.66)	3.95 (0.73)	0.348
Safety behavior	3.92 (0.60)	3.86 (0.53)	3.97 (0.66)	0.163
Health behavior	3.46 (0.84)	3.37 (0.85)	3.54 (0.83)	0.112
Well-being	3.54 (0.62)	3.49 (0.63)	3.59 (0.61)	0.192

^**a**^ Due to missing data, the sample size for safety leadership and well-being was 246 and 245 for health leadership

The 250 participating employees significantly differed from the 2,535 excluded employees based on gender, race and ethnicity, job level, and education. A driving factor in the observed differences was the disproportionate amount of missing data from excluded employees. It is of note that the missingness was mostly due to employees in the excluded group starting the survey but not completing it, rather than purposefully skipping the demographic questions. All baseline demographic comparisons between included and excluded employees can be found in Additional file 1.

A summary of the baseline characteristics of the 250 included in the analysis can be found in Tables 1 and 2. The average employee was 42 years old (SD: 12, Range: 19–71). The majority were white, non-Hispanic (*n* = 217, 87%) and two-thirds worked in non-supervisory roles (*n* = 157, 63%). The average current job tenure was 5 years (SD: 6, Range: 0—33). There were no significant differences between employee gender, age, tenure, education, job level, or baseline scores on all study outcomes based on intervention group.

Table 3 summarizes the observed differences in study outcomes from baseline to follow-up. Businesses improved their TWH policies and programs score from baseline to follow-up, regardless of leadership intervention group assignment. On average, businesses in the intervention group improved their TWH policies and programs score by 16 points (95%CI: -48, 80) compared to an average improvement of 19 points (95%CI: -44, 83) for businesses not in the intervention group (p-value for difference: 0.682). There were no observed differences in employee-rated safety leadership between intervention groups. On average, employee rated safety leadership decreased -0.2 points (95%CI: -1.7, 1.3) from baseline to follow-up in businesses in the intervention group compared to a 0.1-point increase (95%CI: -1.4, 1.6) in businesses not in the intervention group (p-value for difference: 0.121). There were no observed differences in employee-rated health leadership between intervention groups. Regardless of intervention group, average health leadership increased 0.3 points from baseline to follow-up (95%CI: -1.3, 1.9; p-value for difference: 0.455). Similarly, there were no observed differences in safety climate, health climate, safety behaviors, health behaviors, or well-being between those employees whose business participated in the intervention and those who did not.

**Table 3:** Least square mean estimation of study outcomes from baseline to follow-up in the SSWell study stratified by intervention group

		**Intervention**	**No intervention**	
**Business**	**Time**	**Mean (95% CI)**	**Mean (95% CI)**	***p*-value**
TWH policies and programs score	Baseline	41.58 (8.78, 74.37)	43.44 (11.25, 75.63)	0.752
	Follow-up	57.57 (23.69, 91.45)	62.79 (30.08, 95.50)	0.573
	Difference	15.99 (-47.58, 79.57)	19.35 (-43.92, 82.63)	0.682
**Employee**	**Time**	**Mean (95% CI)**	**Mean (95% CI)**	***p*-value**
Safety Leadership	Baseline	3.75 (2.99, 4.51)	3.61 (2.85, 4.37)	0.351
	Follow-up	3.57 (2.76, 4.37)	3.69 (2.94, 4.45)	0.593
	Difference	-0.18 (-1.68, 1.31)	0.09 (-1.38, 1.55)	0.175
Health Leadership	Baseline	3.48 (2.65, 4.30)	3.51 (2.69, 4.33)	0.831
	Follow-up	3.79 (2.92, 4.67)	3.79 (2.98, 4.61)	0.990
	Difference	0.31 (-1.28, 1.92)	0.28 (-1.30, 1.86)	0.880
Safety Climate	Baseline	3.61 (2.94, 4.29)	3.49 (2.83, 4.15)	0.454
	Follow-up	4.09 (3.79, 4.80)	4.10 (3.43, 4.76)	0.986
	Difference	0.48 (-0.81, 1.76)	0.60 (-0.65, 1.86)	0.456
Health Climate	Baseline	4.04 (3.34, 4.74)	4.12 (3.42, 4.81)	0.562
	Follow-up	3.74 (3.00, 4.48)	4.04 (3.34, 4.73)	0.151
	Difference	-0.31 (-1.69, 1.08)	-0.08 (-1.44, 1.28)	0.226
Safety Behavior	Baseline	3.98 (3.46, 4.51)	4.09 (3.57, 4.62)	0.238
	Follow-up	3.75 (3.19, 4.30)	4.02 (3.50, 4.55)	0.076
	Difference	-0.24 (-1.28, 0.81)	-0.07 (-1.10, 0.95)	0.242
Health Behavior	Baseline	3.26 (2.53, 4.00)	3.38 (2.65, 4.12)	0.392
	Follow-up	3.90 (3.12, 4.68)	3.89 (3.16, 4.62)	0.960
	Difference	0.64 (-0.81, 2.09)	0.51 (-0.91, 1.93)	0.495
Well-being	Baseline	3.42 (2.85, 3.99)	3.49 (2.92, 4.06)	0.424
	Follow-up	3.47 (2.87, 4.07)	3.82 (3.25, 4.39)	0.030
	Difference	0.05 (-1.08, 1.19)	0.33 (-0.78, 1.44)	0.071

## Discussion

This study sought to address a gap in the literature regarding small business senior leadership development for TWH. We applied organizational change and leadership theories to intervention science frameworks and developed a TWH leadership intervention that could be applicable to small businesses in any industry. Using a randomized waitlisted control comparison design, we sought to assess changes in business TWH policies and programs as well as employee-reported outcomes, including safety leadership and health leadership practices, safety climate and health climate, safety behaviors and health behaviors, and well-being. We did not observe significant between-group differences in any outcomes from baseline to follow-up and our study is unable to refute the null hypothesis. Our study demonstrates many of the challenges of conducting studies focused on organizational change in workplaces, specifically in small businesses. Below we discuss potential reasons for our null findings as well as the implications for TWH intervention research in small business.

A strength of our study is the representation of diverse small businesses from multiple industries and urban and rural geographical regions and initial success in recruitment and enrollment of almost one hundred small businesses to study the broad applicability of a TWH leadership development program to small businesses across industry sectors. To date, much of the TWH intervention research literature has focused on employee-level interventions in a single business or a small number of companies in a single sector [8, 41]. Prior to our study, we established relationships with several intermediaries that work directly with small businesses [4, 34]. Leveraging an existing, and trusted, platform such as Health Links to recruit businesses proved successful. In addition, our strategies to reach small employers by presenting at local industry and professional meetings, promotion in partner organization communications, and referrals from local contacts yielded positive reach and engagement among our target audiences.

Despite recruiting almost one-hundred businesses, this may not have been enough to address our study questions. Our a-priori power calculation for study recruitment assumed a 10-point change in TWH policies and programs score (SD: 20) in the intervention group, and no change in the non-intervention group requiring 128 businesses to have complete data. Thus, we were not successful in recruiting the number of businesses needed to address our research question. Additionally, we struggled to retain businesses in the study. We observed a significant loss to follow-up with only 37% of businesses remaining in the study at follow-up, despite frequent “touch points”. Small businesses face many pressures. The retention challenges that we faced become even more concerning when we consider that businesses which initially enrolled may be among the more motivated and engaged employers. A limitation of our study is that the companies that were retained may be particularly enthusiastic about TWH, limiting generalizability of findings. Our study outcomes were based on self-reported data and thus may be biased if respondents replied in ways that attempted to make themselves or their organizations appear in a favorable light. Thus, the combination of recruitment and retention challenges may have resulted in an under powered study, and this may provide one possible explanation for our null results.

Of possibly greater concern for research in the small business arena is that we especially struggled to convince small business leaders to devote enough of their own time and effort to participate in the intervention at a level needed to be considered an effective training experience. Usually, our main corporate contact had to get buy-in from the senior leader to attend the training. Even when leaders agreed to participate, on the day of training, several barriers harmed attendance, including inclement weather, competing business priorities, urgent developments at work, and personal illness. This resulted in a smaller final sample size in the intervention group than was anticipated.

Organizations that ultimately were retained were larger and reported more TWH policies and programs at baseline than did non-participating employers. Prior research in small business TWH has stressed the importance of interventions that are inexpensive, not resource/time intensive, and integrated into their existing business functions [30]. We know that the smallest of employers often have a limited market share, high resource constraints, and can operate under extreme financial pressure with a high potential for failure [42]. We posit those employers under these pressures may have had a hard time re-enrolling in the study or scheduling their leadership for the TWH leadership development program. Our findings suggest that TWH interventions which place high demands on small business leaders’ time may not be feasible. More knowledge is needed to understand what training methods would be most appealing for small business leaders. Researchers will need to consider a balance between intervention intensity and leader participation and retention.

We hypothesize that we either missed the intervention effect by not measuring our study outcomes earlier than one year or we did not wait long enough. While we may not have observed transactional or transformational changes as expressed through our business and employee assessments, we know the intervention was effective at short-term (3-month) change in self-reported leadership behaviors. In a prior study, we observed the direct effects of the leadership intervention on leader-reported behaviors within three months [22]. Our study timeline for measuring changes to the business and employee outcomes from baseline to one year may have been too short and contributed to our null findings. For businesses that participated in the leadership training, the follow-up business and employee assessments were conducted on average 6 months post training but ranged from 5 to 300 days. This indicates that there was variation in the time between completion of the intervention and follow-up assessments to evaluate the intervention. Some businesses may not have had enough time to make changes after the intervention. Researchers note that the interval from which to measure leadership intervention effects is unclear [6]. Our program evaluation results indicated that leaders were commonly working on foundational goals related to TWH policy and program development that may need time to implement [22]. In contrast to our intervention, most other TWH-related and safety-specific intervention studies that include leadership focus on supervisors and changes in outcomes amongst their teams within a few weeks or months with mixed findings [12, 13, 15, 16, 18, 19, 43–46].

The retention of employees in follow-up surveys may play heavily into decisions about when study outcomes are measured. The longer the follow-up timeframe the more challenging it is to obtain data from the same workers and this will vary by industry [47]. Some of our loss to follow-up may have been due to our methods of data collection. Had we contacted employee study participants directly during follow-up, instead of emailing them via our main contact at each organization, we might have had a higher response rate. However, given the scale of this study, it was not feasible for us to do this. In other studies, we have provided periodic updates about the study progress and results to the participants to promote continued engagement. However, employers proved unwilling to allow our research team to directly communicate or contact their employees, which, we speculate, may have weakened employee perception of the importance of their continued participation in the research.

### Future research

Ultimately, our study was unable to determine if leadership training on TWH can produce transformational and transactional changes in small businesses that are sufficient to enhance TWH adoption and improve safety, health and well-being of workers. We learned that there are substantial obstacles to answering this important question, including how to secure enough of the time and attention of busy small business leaders to deliver an effective TWH leadership program. Future research must grapple with the calculus of the business owner’s available time for leadership training and the minimum effective dose of a leadership intervention. Studies of small business owners suggest that when it comes to occupational safety and health, they often allow other priorities to take precedence, vary in their levels of hazard risk perception, feel obligated to focus on immediate programmatic issues rather than their leadership skills, and tend to want to share responsibility with others [29, 48, 49]. One potential method to engage this audience may be to frame the intervention as part of a larger effort to create shared leadership for TWH in their business. This strategy, akin to TWH participatory intervention approaches [50], would focus on sharing responsibility and influence for TWH [51].

The mechanisms by which TWH leadership interventions impact organizational and employee outcomes warrant further investigation. In the present study, we assessed the direct effect of the leadership program on our outcomes of interest. However, it is likely that there are underlying mechanisms by which a leadership program results in changes to our outcomes of interest. For example, the leadership program should lead to changes in leader behavior that would in turn lead to changes in climate perceptions. Although our experience suggests that it will be logistically daunting and expensive, larger small business cohorts and more time for generating longitudinal data will be required to assess these mechanistic questions.

## Conclusions

When designing TWH intervention studies, researchers should consider how to best engage small business leaders in interventions and implementations early on, as well as methods that are well matched to measuring primary and secondary outcomes longitudinally. Future research is needed to test the feasibility and sustainability of TWH interventions in small business.

## Supplementary Information

**Additional file 1. **Employer and employee characteristics between those who were included in the final study population and those who were not. This table compares the demographic characteristics of employers and employees who were included in the final study population and those who were not.

## Data Availability

The dataset analyzed during the current study are not publicly available due the fact that we are still currently analyzing and publishing with the dataset but are available from the corresponding author on reasonable request.

## Abbreviations

TWH: Total Worker Health
Small + Safe + Well: SSWell
REDCap: Research Electronic Data Capture
