# Supplementary material for: Small + Safe + Well: lessons learned from a Total Worker Health® randomized intervention to promote organizational change in small business
Source: BMC Public Health. 2022 May 24;22:1039. doi: 10.1186/s12889-022-13435-y (PMC9128251; doi:10.1186/s12889-022-13435-y)
Supplement: Supplementary file 1 — Additional file 1. Employer and employee characteristics between those who were included in the final study population and those who were not. This table compares the demographic characteristics of employers and employees who were included in the final study population and those who were not. [file 12889_2022_13435_MOESM1_ESM.pdf]

Additional File 1. Employer and employee characteristics between those who were included in the final study population and those who were not.

Employer Level Characteristics			
	Included N = 36	Excluded N = 61	p-value
Industry			0.439
Construction	3 (8%)	2 (3%)	
Education	3 (8%)	3 (5%)	
Healthcare/Social assistance	10 (28%)	11 (18%)	
Public Admin	2 (6%)	5 (8%)	
Other	18 (50%)	40 (66%)	
Size			0.262
Micro (< 11)	3 (8%)	11 (18%)	
Small (11 - 50)	13 (36%)	28 (46%)	
Medium (51 - 200)	14 (39%)	15 (25%)	
Large (201+)	6 (17%)	7 (12%)	
Total Number of Employees	109 (113)	61 (75)	0.014
Baseline Heath Links Total Score	46 (18)	37 (14)	0.005
Individual Level Characteristics			
	Included N = 250	Excluded N = 2535	p-value
Age	42 (12)	41 (13)	0.0506
Female	186 (74%)	1415 (65%)	0.014
Race/ethnicity			<0.001
White, non-Hispanic	217 (87%)	1708 (67%)	
Hispanic/Latino/Spanish Origin	24 (10%)	314 (12%)	
Black/African American	2 (1%)	39 (2%)	
Asian	2 (1%)	26 (2%)	
Native American/Alaskan Native	1 (0%)	14 (1%)	
Native Hawaiian/Pacific Islander	0 (0%)	4 (0%)	
Multiracial	3 (1%)	50 (2%)	
Did not provide	1 (0%)	380 (15%)	
Job level			<0.001
Manager	92 (37%)	877 (35%)	
Non-manager	157 (63%)	1291 (51%)	
Did not provide	1 (0%)	367 (15%)	
Tenure, years	5 (6)	6 (7)	0.853
Education			<0.001
Did not complete high school	0 (0%)	653 (26%)	
High school/GED	20 (8%)	237 (9%)	
Some college/2-year degree	62 (25%)	494 (20%)	
4-year college degree	104 (42%)	736 (29%)	
Graduate/professional degree	54 (22%)	397 (16%)	
Did not provide	10 (4%)	653 (26%)	
